# LKB1 is essential for the proliferation of T-cell progenitors and mature peripheral T cells

**DOI:** 10.1002/eji.200939677

**Published:** 2010-01

**Authors:** Peter Tamás, Andrew Macintyre, David Finlay, Rosemary Clarke, Carmen Feijoo-Carnero, Alan Ashworth, Doreen Cantrell

**Affiliations:** 1 Division of Cell Biology and Immunology, School of Life Sciences, University of DundeeDundee, Scotland, United Kingdom; 2 The Breakthrough Breast Cancer Centre, the Institute of Cancer ResearchLondon, England, United Kingdom

**Keywords:** LKB1, Metabolism, T-cell development

## Abstract

The serine/threonine kinase LKB1 has a conserved role in *Drosophila* and nematodes to co-ordinate cell metabolism. During T lymphocyte development in the thymus, progenitors need to synchronize increased metabolism with the onset of proliferation and differentiation to ensure that they can meet the energy requirements for development. The present study explores the role of LKB1 in this process and shows that loss of LKB1 prevents thymocyte differentiation and the production of peripheral T lymphocytes. We find that LKB1 is required for several key metabolic processes in T-cell progenitors. For example, LKB1 controls expression of CD98, a key subunit of the l-system aa transporter and is also required for the pre-TCR to induce and sustain the regulated phosphorylation of the ribosomal S6 subunit, a key regulator of protein synthesis. In the absence of LKB1 TCR-β-selected thymocytes failed to proliferate and did not survive. LBK1 was also required for survival and proliferation of peripheral T cells. These data thus reveal a conserved and essential role for LKB1 in the proliferative responses of both thymocytes and mature T cells.

## Introduction

T-cell proliferation and differentiation in the thymus and the periphery are regulated by the pre-TCR, the mature TCR, cytokines and chemokines 1–3. These stimuli are linked *via* tyrosine kinases to a diverse network of serine/threonine kinases that regulate the key checkpoints of T-cell proliferation and differentiation 4–6. T-cell expansion in the thymus is an energy-demanding process that only proceeds when extra cellular signals from Ag receptors, cytokines and stromal cells stimulate sufficient cellular energy production and nutrient uptake to satisfy the biosynthetic demands of the activated T cell 7–9. For example, during T-cell development in the thymus there is rapid proliferative expansion of TCR-β selected T-cell progenitors 3. To meet the increased energy demands of these proliferating cells, the pre-TCR and Notch induce and then maintain cell surface expression of nutrient receptors such as aa transporters and transferrin receptor and also increase the expression of the glucose transporter. These increases in glucose metabolism and aa uptake are essential for T-cell development in the thymus. For example, the serine/threonine kinase phosphoinositide dependent kinase 1 (PDK1) and its substrates protein kinase Bα (PKBα), β and γ regulate the expression of glucose and aa transporters in thymocytes. T-cell progenitors that do not express PDK1 or that lack expression of PKB isoforms fail to express these nutrient receptors and fail to develop because they cannot meet the metabolic demands of thymus development 7,8,10,11.

One other serine/threonine kinase that can regulate cellular responses to energy stress is LKB1 (or serine/threonine kinase 11 -STK11) 12. This is an evolutionarily conserved kinase: Par4, the *Caenorhabditis elegans* ortholog, is one of the six “partitioning” molecules that control zygote polarity 13 in *Drosophila*. The LKB1 ortholog is required to synchronize cellular energy checkpoints and cell division 14,15. The *Drosophila* LKB1 homologue is thus essential for mitotic spindle formation, for the establishment of cell polarity and controlling the asymmetric division of stem cells 16. LKB1 also has essential functions in mice as LKB1 deletion causes problems with vascular and neural development that result in embryonic lethality at E10-11 17. In humans the importance of LKB1 is highlighted by the fact that it is mutated in a high proportion of Peutz-Jeghers syndrome patients: Peutz-Jeghers syndrome is associated with the development of benign hamartomas and an increased risk of malignant tumor formation 18–20. LKB1 is important because it phosphorylates critical activating residues in the catalytic domains of multiple members of the AMP-activated protein kinase (AMPK) family including the α1 and α2 isoforms of AMPK and NUAK1–2, BRSK1-2, QIK, QSK, Salt-inducible kinase (SIK), MELK and MARK1–4 kinases 21. The AMPKα1 and α2 are phosphorylated and activated by LKB1 in response to increases in cellular AMP:ATP ratio. AMPK then act to restore energy balance in a cell by inhibiting ATP consuming processes and stimulating ATP generating pathways 22. SIK and MARK2 also regulate cellular metabolic responses in different tissues leading to a model whereby LKB1 acts to regulate the energy status of the cell 23–25. The significance of LKB1 in energy checkpoints is illustrated by the fact that loss of LKB1 in fibroblasts and in the pancreas is associated with apoptosis in response to energy stress 26,27. There is also evidence that LKB1 controls the induction of autophagy in response to energy deprivation and sensitizes epithelial cells to c-myc-induced apoptosis 28,29.

The role of LKB1 and AMPK family kinases in lymphocytes is not known but is topical because of the increasing awareness that energy control and the regulation of asymmetric cell division may control T lymphocyte fate 11,30. In mature T cells, the α1 AMPK isoform is expressed and is activated by TCR triggering *via* calcium/calmodulin dependent kinase kinases and by energy stress, presumably *via* LKB1 31. AMPK α1-null T cells have increased sensitivity to energy stress but can mount apparently normal immune responses 32. There are, however, immune defects in mice that lack expression of another AMPK family kinase, MARK2. T cells lacking expression of MARK2 are hyper-responsive to TCR triggering with increased cytokine production and MARK2-null mice develop autoimmune disease, indicating that MARK2 is required for immune homeostasis 33. Interestingly, neither AMPkα1- nor MARK2-null mice have defects in T-cell development in the thymus 32,33. This could indicate that AMPK family kinases are physiologically irrelevant for T-cell development but it is perhaps more likely that there is redundancy between different AMPK family members. One experimental strategy that circumvents problems caused by the existence of multiple redundant kinases is to delete a rate-limiting specific upstream regulator. In the context of the AMPK family the obvious candidate is LKB1, a “master kinase” that phosphorylates and activates multiple AMPK family kinases 21. Accordingly, the present study reports the impact of T-lineage-specific deletion of LKB1 and shows that LKB1 has essential functions for the development of TCR-β selected T-cell progenitors. LBK1 was also required for survival and proliferation of peripheral T cells revealing that AMPK family kinases control proliferative checkpoints in thymocyte differentiation and in peripheral T cells.

## Results

### Deletion of LKB1 prevents T-cell development

**Figure 1:**
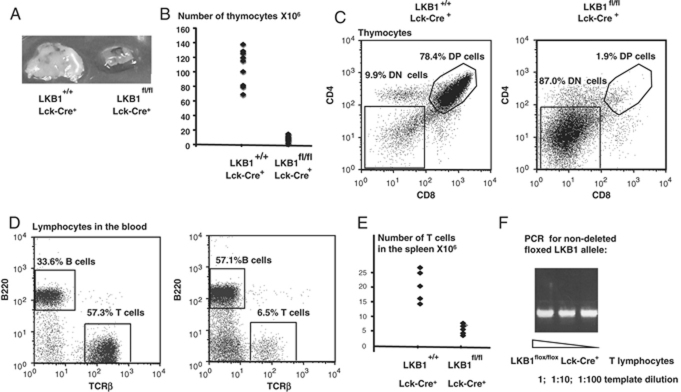
Deletion of LKB1 prevents T-cell development (A) Appearance of thymi in LckCre^+^LKB1^fl/fl^ and LckCre^+^LKB1^+/+^ mice. (B) Total thymocyte numbers from 5- to 8-wk-old LckCre^+^LKB1^fl/fl^ (*n*=10) and LckCre^+^LKB1^+/+^ (*n*=10) mice. (C) Flow cytometry of CD4 and CD8 subpopulations in thymocytes from LckCre^+^LKB1^fl/fl^ and LckCre^+^LKB1^+/+^ mice. Dot plots of Thy-1-gated cells are representative of ten independent experiments. (D) Flow cytometry of T cell (TCRβ^+^) and B cell (B220^+^) subpopulations in blood from LckCre^+^LKB1^fl/fl^ and LckCre^+^LKB1^+/+^ mice. Dot plots are representative of three independent experiments. Gates show T and B cell populations. (E) Total numbers of T lymphocytes in spleens from LckCre^+^LKB1^fl/fl^ (*n*=5) and LckCre^+^LKB1^+/+^ (*n*=5) mice. (F) Genomic PCR analysis of the fl/fl LKB1 gene in T lymphocytes from spleen indicating that peripheral T cells have not deleted the LKB1 allele.

The objective of the present study was to explore the role of LKB1 in T lymphocytes. Mice with floxed LKB1 exons 4–7 on both alleles (LKB1^fl/fl^) 34 were backcrossed with mice expressing Cre recombinase under the control of the proximal p56lck proximal promoter (Lck-Cre^+^), which induces Cre expression in T-cell progenitors in the thymus 35. The LckCre^+^LKB1^fl/fl^ mice were viable, although they had very small thymi (Fig. 1A) that lacked the normal cortical/medullar architecture (data not shown). These small LckCre^+^ LKB1^fl/fl^ thymi contained greatly reduced numbers of thymocytes compared to LckCre^+^LKB1^+/+^ controls (Fig. 1B). To determine which stages of thymocyte development were sensitive to LKB1 loss, LckCre^+^LKB1^fl/fl^ thymi were analyzed for expression of the major histocompatibility complex (MHC) receptors CD4 and CD8. Early T-cell progenitors are double negative (DN) for CD4 and CD8. DN progenitors undergo TCR-β locus rearrangements to produce a TCR-β polypeptide that permits surface expression of the pre-TCR complex. The pre-TCR then supports survival and directs rapid clonal expansion along with differentiation of cells into CD4^+^CD8^+^ double positive (DP) thymocytes. TCR α-chain gene rearrangements then occur and cells that express a functional but non self reactive α/β TCR complex differentiate to either CD4^+^ or CD8^+^ single positive (SP) T cells 1,3,36. Figure 1C shows that LckCre^+^LKB1^fl/fl^ thymi contained mostly DN cells and had very few DP or SP. There were also very few peripheral T cells present in the blood or spleen of the LckCre^+^LKB1^fl/fl^ mice compared to LckCre^+^ LKB1^+/+^ control animals (Fig. 1D and E). Moreover, in the few peripheral T cells of LckCre^+^LKB1^fl/fl^ mice there was evidence that LKB1 deletion was not complete, as cells contained non-deleted LKB1 floxed alleles (Fig. 1F). Initial analysis of LckCre^+^LKB1^fl/fl^ mice thus showed that LKB1 is essential for T-cell development in the thymus; only cells that fail to delete LKB1 can differentiate in the thymus and generate peripheral T cells.

### DN3/DN4 transition defects in LKB1-null thymocytes

**Figure 2:**
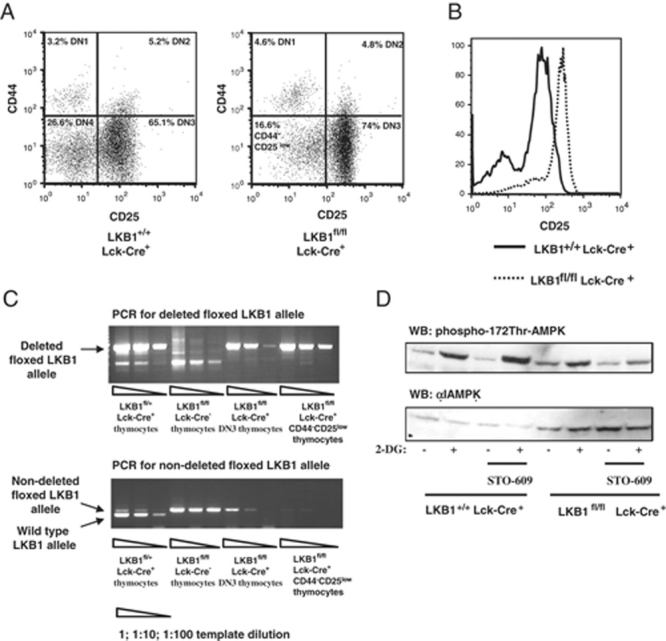
DN3/DN4 transition defects in LKB1-null thymocytes. (A) Flow cytometry of CD44 and CD25 surface expression in CD4^−^CD8^−^ DN thymocytes from LckCre^+^LKB1^fl/fl^ and LckCre^+^LKB1^+/+^ thymi. CD25 and CD44 profiles of Thy-1^+^ DN cells additionally gated to exclude cells from the non-TCR lineage (lineage). Gates show DN1-DN4 populations, representative of five independent experiments. (B) Surface expression of CD25 in lineage Thy-1^+^ thymocytes from LckCre^+^LKB1^fl/fl^ and LckCre^+^LKB1^+/+^ mice, representative of five independent experiments. (C) Genomic PCR analysis of the LKB1 gene in DN3 and CD44^−^CD25^low^ thymocytes. Top, PCR amplification of the gene product produced only when the LKB1 floxed allele is deleted in Lck-Cre^+^ thymocytes. Bottom, PCR amplification of the non-deleted LKB1 gene. The upper bands represent the PCR product generated from the LKB1 non-deleted floxed gene, while the lower band indicates the WT LKB1 gene. (D) LckCre^+^LKB1^fl/fl^ CD44^−^CD25^low^ thymocytes and LckCre^+^LKB1^+/+^ DN4 thymocytes were either untreated or pretreated with STO-609 and then stimulated with 50 mM 2-deoxyglucose for 5 min. Western blot of cell lysates prepared from these thymocytes with pThr-172–AMPK and AMPK α1 antisera, representative of three independent experiments.

DN thymocytes can be subdivided on the basis of CD44 and CD25 expression: the earliest progenitors are CD44^+^and CD25^−^ DN1), followed sequentially by the CD44^+^ and CD25^+^ (DN2), CD44^−^ and CD25^+^ (DN3) and CD44^−^CD25^−^ DN4 ) populations 3,36. CD25 and CD44 staining profiles showed that LckCre^+^LKB1^fl/fl^ thymi had an increased frequency of the DN3 subset: numbers of DN3 thymocytes were 4.0–9.1×10^6^ in LckCre^+^LKB1^+/+^ while 3.6–7.6×10^6^ in LckCre^+^LKB1^fl/fl^ thymi (Fig. 2A). Moreover, DN3 cells in LckCre^+^LKB1^fl/fl^ thymi showed abnormally high levels of CD25 expression (Fig. 2B). There was no subset of cells corresponding to WT DN4, *i.e.* CD44^−^CD25^−^ in LckCre^+^ LKB1^fl/fl^ thymi although there was a population of CD44^−^ CD25^low^ cells that resembled cells in transition from the DN3 to DN4 stage. Numbers of DN4 thymocytes were 1.6–3.7×10^6^ in LckCre^+^LKB1^+/+^ thymi while the numbers of CD44^−^CD25^low^ thymocytes were 3.6–7.6 and 0.8–2.1×10^6^ in LckCre^+^LKB1^fl/fl^ samples. The first phenotypic difference between LckCre^+^LKB1^fl/fl^ and normal thymi is thus seen in DN3 thymocytes; some cells partially transit to the DN4 stage albeit at lower frequency than normal. The proximal p56Lck promoter is active in DN1 cells, although its activation tends to be heterogeneous until the DN3 stage 37. In this context, genomic PCR analysis indicated partial deletion of LKB1 in DN3 cells and complete loss in the CD44^−^ CD25^low^ cells (Fig. 2C).

LKB1 is known to phosphorylate and activate AMPK in response to energy stress (22,38. Therefore to assess functional loss of LKB1, we compared the ability of 2-deoxyglucose, an inhibitor of glycolysis that increases cellular ratios of AMP/ATP, to activate AMPK in control and LckCre^+^LKB1^fl/fl^ DN thymocytes. AMPKα1 can also be phosphorylated and activated by calcium/calmodulin dependent kinase kinases (CaMKK) in T cells 31 so the effect of the CaMKK inhibitor STO609 on AMPK activation was also examined. Figure 2D shows that there is low basal phosphorylation of Thr 172 in the activation loop of AMPKα1 in WT pre-T cells and that this phosphorylation is increased by 2-DG treatment. AMPKα1 Thr 172 phosphorylation was not inhibited by STO609. Basal phosphorylation of AMPKα1 in LckCre^+^LKB1^fl/fl^ thymocytes was observed, but this was not increased by 2-DG treatment.

### LKB1 and pre-TCR/Notch signaling

**Figure 3:**
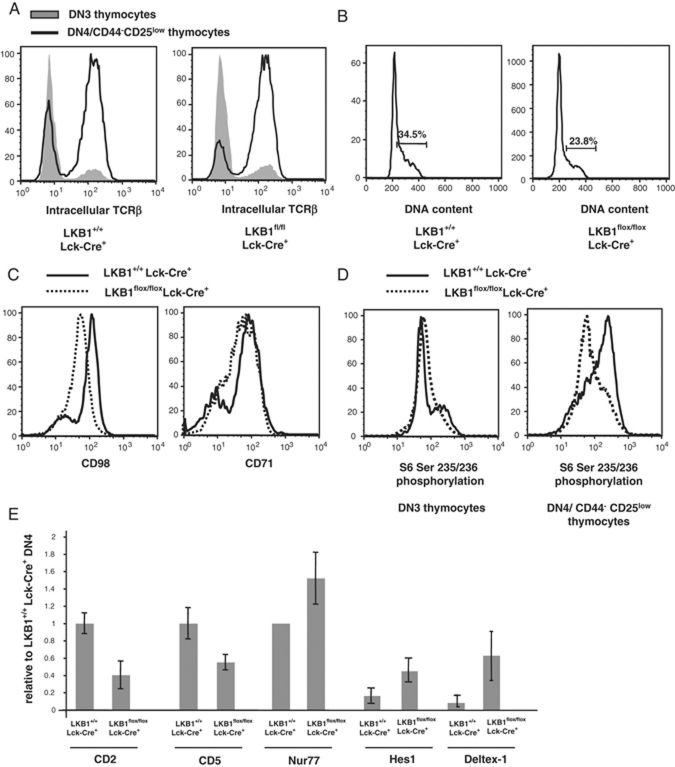
PreTCR and Notch signaling in LKB1-null thymocytes (A) Intracellular TCRβ expression in DN3 and DN4 thymocytes of LckCre^+^LKB1^+/+^ and DN3 and CD44^−^CD25^low^ thymocytes of LckCre^+^LKB1^fl/fl^ mice. (B) Flow cytometry of cellular DNA content (gated on live cells) of DN4 thymocyte from LckCre^+^LKB1^+/+^ and CD44^−^CD25^low^ thymocytes from LckCre^+^LKB1^fl/fl^ mice. The numbers indicate percentage of cells in S+G2/M phases of the cell cycle, representative of three independent experiments. (C) Data show CD98 and CD71 surface expression on DN4 thymocytes from LckCre^+^LKB1^+/+^ and on CD44^−^CD25^low^ thymocytes from LckCre^+^LKB1^fl/fl^ mice, representative of three independent experiments. (D) Histograms show Ser 235/236 S6 ribosomal protein phosphorylation in DN3 and DN4 thymocytes from LckCre^+^LKB1^+/+^and in DN3 and CD44^−^CD25^low^ thymocytes from LckCre^+^LKB1^fl/fl^ mice, representative of three independent experiments. (E) Quantitative RT-PCR examining the expression levels of CD2, CD5, Nur77, Hes1 and Deltex1 mRNA in DN4 thymocytes of LckCre^+^LKB1^+/+^ (*n*=3) and CD44^−^CD25^low^ thymocytes from LckCre^+^LKB1^fl/fl^ (*n*=5) mice. Expression was normalized to the mRNA levels in LKB^+/+^ Lck-Cre^+^ DN4 thymocytes; data show mean±SD. Differences in CD2 and CD5 expression are considered to be significant (*p*=0.035 and *p*=0.036 for CD2 and CD5, respectively, *p*=0.1 for Nur77).

CD25 downregulation in DN3 is driven by the pre-TCR 39. The high level of CD25 on LckCre^+^LKB1^fl/fl^ DN3 cells coupled with the failure of these cells to completely downregulate CD25 and complete transit to DN4 cells could thus reflect a problem with pre-TCR expression or function. The rate-limiting step for pre-TCR expression is normally the TCR β locus rearrangement 40. However, analysis of intracellular TCR-β expression showed that LckCre^+^LKB1^fl/fl^ DN3 cells had successfully rearranged their TCR-β locus and expressed intracellular TCR subunits at the normal frequency (approximately 15%) (Fig. 3A). It was also seen that the subpopulation of LKB1-null pre-T cells that had partially downregulated CD25 were predominantly TCR-β selected (80%) and in this regard were indistinguishable from bona fide WT DN4 (Fig. 3A).

One difference between WT and LKB1 deleted DN3 cells is that the latter have increased expression of CD25, a phenotype that frequently results from defective pre-TCR signal transduction. We therefore examined a range of pre-TCR-induced responses in LckCre^+^LKB1^fl/fl^ pre-T cells. One key function of the pre-TCR is to drive cell cycle progression as cells transit from DN3 to DN4. Flow cytometric analysis of cellular DNA content showed that LKB1 loss did not block cell cycle entry, as LKB1-null pre-T cells can enter the proliferative S/G2 phases of the cell cycle albeit at a reduced frequency compared to controls (Fig. 3B). Pre-TCR signaling also induces expression of key nutrient receptors on T-cell progenitors, namely CD71 the transferrin receptor and CD98, a subunit of the l-aa transporter 7. LKB1 loss had no impact on surface expression of CD71 the transferrin receptor but there was a striking decrease in the surface expression of CD98 aa transporter in LckCre^+^LKB1^fl/f^ pre-T cells (Fig. 3C).

We also analyzed the effect of LKB1 deletion on pre-TCR-induced phosphorylation (Ser235/236) of the S6 ribosomal subunit by the 70 kDa ribosomal S6 kinases. S6 phosphorylation can be quantified directly *ex vivo* by flow cytometry and intracellular staining of fixed cells with specific phospho-S6 antisera 10. WT DN3 thymocytes analyzed immediately *ex vivo* are heterogeneous for phosphoS6: the majority of cells are phosphoS6^low^ but 15–20% are phosphoS6^high^ corresponding to cells that have rearranged their TCR-β subunit to express the pre-TCR. DN4 thymocytes are primarily phosphoS6^high^ (Fig. 3D). The data show severely reduced phosphorylation of S6 in LckCre^+^LKB1^fl/fl^ stained DN thymocytes. S6 phosphorylation is induced by PDK1/PKB signaling pathways initiated by the pre-TCR and sustained by stromal signals such as Notch ligands 41. In this respect, LKB1-null T cells had normal expression of CD71, the transferrin receptor. This is relevant because CD71 expression in T-cell progenitors is controlled by pre-TCR/Notch-induced PDK1/PKB-mediated pathways 7. The normal expression of CD71 is thus evidence for functioning PKB signaling in LKB1-null pre-T cells. We further explored the impact of LKB1 on pre-TCR signaling by examining expression of known pre-TCR-induced genes, such as CD2 and CD5 and Nurr77 7,42,43. LKB1-null DN4 pre-T cells expressed lower levels of CD2 mRNA than controls and there was also a small but reproducible decrease in expression of CD5 mRNA. However, the expression of Nur77, also a pre-TCR-induced gene, was not changed (Fig. 3E). The results argue that LKB1 loss causes selective defects but not global problems with pre-TCR signal transduction. We also examined the expression of Notch target genes, Hes-1 and Deltex-1^44^, in pre-T cells isolated *ex vivo* from control or LckCre^+^LKB1^fl/fl^ mice. The CD44^−^CD25^low^ cells from LckCre^+^LKB1^fl/fl^ mice expressed increased levels of Hes-1 and Deltex1 when compared with control cells (Fig. 3E). The expression of these Notch target genes in LKB1-null pre-T cells argues that these cells are responding to Notch ligands *in vivo*.

### LKB1 is required for survival of TCR-β selected T-cell progenitors

**Figure 4:**
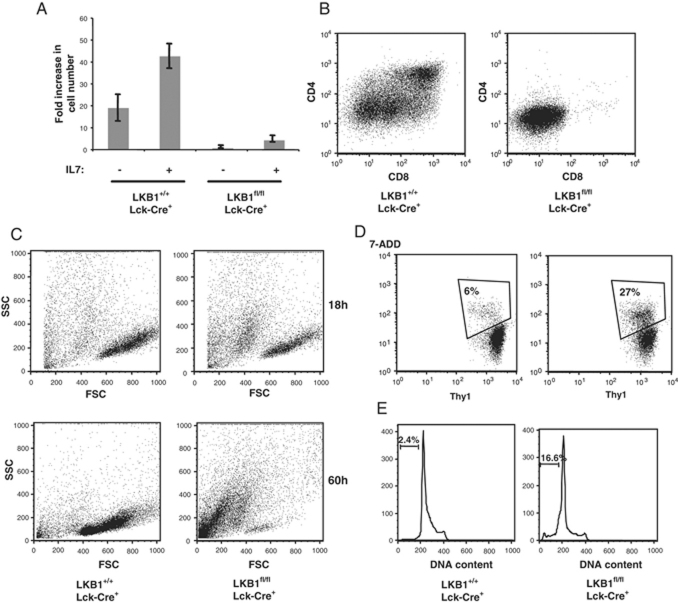
LKB1 is required for survival of TCRβ selected T-cell progenitors A) DN thymocytes from LckCre^+^LKB1^+/+^ (*n*=3) and LckCre^+^LKB1^fl/fl^ (*n*=3) mice were co-cultured with OP9-DL1 stromal cell monolayers. Cells were either stimulated with IL-7 or left untreated. Data show the mean fold increase in cell number following 5 days culture±SD. Differences in the proliferation of LckCre^+^LKB1^+/+^ and LckCre^+^LKB1^fl/fl^ DN thymocytes are considered to be significant (*p*=0.007 without IL-7 and *p*=0.0005 in the presence of IL-7, respectively). The IL-7-induced proliferation of LckCre^+^LKB1^fl/fl^ DN thymocytes is also significant, *p*=0.004. (B) DN thymocytes from LckCre^+^LKB1^+/+^ and CD44^−^CD25^low^ thymocytes from LckCre^+^LKB1^fl/fl^ mice were co-cultured with OP9-DL1 stromal cell monolayers and treated with IL-7. The surface expression of CD4 and CD8 on thymocytes and the cell number were analyzed after 5 days of co-culture, representative of three independent experiments. C) DN4 thymocytes from LckCre^+^LKB1^+/+^ and CD44^−^CD25^low^ thymocytes from LckCre^+^LKB1^fl/fl^ mice were co-cultured with OP9-DL1. Flow cytometric analysis of forward and side scatter after 18 and 60 h of co-culture, respectively. (D) DN4 thymocytes from tamoxifen LckCre^+^LKB1^+/+^ and CD44^−^ CD25^low^ thymocytes from LckCre^+^LKB1^fl/fl^ mice were co-cultured with OP9-DL1 for 18 h, and stained with Thy1 and 7-aminoactinomycin D to investigate cell death. The numbers indicate the percentage of dead thymocytes. E) DN4 thymocytes from LckCre^+^LKB1^+/+^ and CD44^−^ CD25^low^ thymocytes from LckCre^+^LKB1^fl/fl^ mice were co-cultured with OP9-DL1 stromal cell monolayers. The data show the DNA content of DN4 thymocytes (not gated, total thymocyte population) after 18 h of co-culture. The numbers show the percentage of cells with degraded DNA.

The differentiation and proliferation of β selected pre-T cells in the thymus is dependent on sustained Notch receptor/ligand interactions 8,41,45. To explore further impact of LKB1 deletion on thymocyte development, we compared the responses of pre-T cells from control or LckCre^+^LKB1^fl/fl^ mice in an *in vitro* system that uses OP9 stromal cells expressing the Notch ligand delta-like 1 (OP9-DL1) and IL-7 to support survival, proliferation and differentiation of T-cell progenitors 46. In initial experiments we compared the responses of cells maintained on the OP9-DL1 cells in both the presence and absence of IL-7. As described previously, normal DN thymocytes (LckCre^+^LKB1^+/+^) undergo proliferative expansion when cultured on OP9-DL1 cells and this proliferative response is enhanced by the addition of IL-7 47 (Fig. 4A). In contrast, LKB1-null DN thymocytes (LckCre^+^LKB1^fl/fl^) did not proliferate on OP9-DL1 cells. The addition of IL-7 to the cultures caused a small improvement to the proliferative response of the LKB1-null DN thymocytes, indicating that the cells had not lost the capacity to respond to IL-7 but the cells still failed to undergo proliferative expansion in these *in vitro* models. Moreover, the proliferative expansion of normal TCR-β selected DN4 (LckCre^+^LKB1^+/+^) is followed by their differentiation to DP thymocytes. Strikingly, TCR-β selected CD44^−^CD25^low^LckCre^+^LKB1^fl/fl^ thymocytes do not differentiate to DP on OP9-DL1 cells (Fig. 4B), in fact they die. LKB1-null pre-T cells thus rapidly acquire the light scattering profiles of dead cells rather than viable cells (Fig. 4C). LKB1-null pre-T cells also show a rapid decrease in plasma membrane integrity (Fig. 4D) and a high frequency of cells with a sub-G1 DNA content, indicative of DNA degradation (Fig. 4E). LKB1 is thus essential for the survival of TCR-β selected T-cell progenitors as they proliferate and differentiate in response to Notch ligands and IL-7.

### LKB1 is required for survival of proliferating peripheral T cells

**Figure 5:**
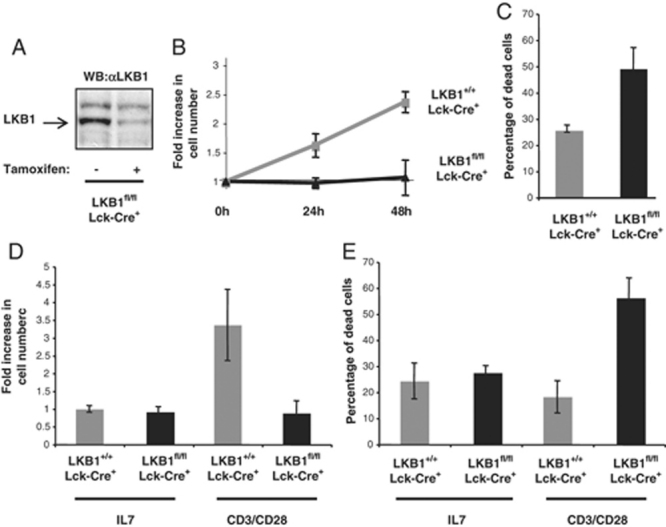
LKB1 is required for survival of proliferating peripheral T cells. (A) LKB1 Western blot of cell lysates from *CreER^T2^*LKB1^fl/fl^ mouse T lymphoblasts either treated or not with 0.4 μM 4-hydroxytamoxifen for 3 days. (B) Fold increase in cell number of T lymphoblasts from *CreER^T2^*LKB1^+/+^ and *CreER^T2^*LKB1^fl/fl^ mice. The cells were treated with 0.4 μM 4-hydroxytamoxifen for 3 days prior to the start of analyses. Data show mean±SD. Differences in the proliferation of LckCre^+^LKB1^+/+^ and LckCre^+^LKB1^fl/fl^ lymphoblasts are considered to be significant (*p*=0.001 at 24 h and *p*=0.0005 at 48 h). (C) Percentage of dead cells (based on FSC/SSC profile) in the 4-hydroxytamoxifen treated T lymphoblast cultures from *CreER^T2^*LKB1^+/+^ and *CreER^T2^*LKB1^fl/fl^ mice. The data corresponds to the 48 h time point in Fig. 5B. Data show mean±SD. Differences between LckCre^+^LKB1^+/+^ and LckCre^+^LKB1^fl/fl^ samples are considered to be significant (*p*=0.003). (D) Primary T cells from *CreER^T2^*LKB1^+/+^ and *CreER^T2^*LKB1^fl/fl^ mice were either treated with IL-7 or stimulated with CD3/CD28 Ab-coated beads. The cells were treated with 0.6 μM 4-hydroxytamoxifen for 4 days prior to stimulation. Data show fold increase in cell number following 2 days of stimulation. Data show mean±SD. Differences between CD3/CD28 stimulated LckCre^+^LKB1^+/+^ and LckCre^+^LKB1^fl/fl^ cells are considered to be significant (*p*=0.002). For IL-7 treated samples, *p*=0.15. (E) Naïve T cells from *CreER^T2^*LKB1^+/+^ and *CreER^T2^*LKB1^fl/fl^ mice were either treated with IL-7 or stimulated with CD3/CD28 Ab-coated beads. The cells were treated with 0.6 μM 4-hydroxytamoxifen for 4 days prior to the stimulation. Data shows the percentage of dead cells (based on FSC/SSC profile) following 2 days of stimulation. Data show mean±SD. Differences between CD3/CD28 stimulated LckCre^+^LKB1^+/+^ and LckCre^+^LKB1^fl/fl^ cells are considered to be significant (*p*=0.00004). For IL-7 treated samples, *p*=0.3.

Does this requirement for LKB1 support the proliferation of T-cell progenitors unique to this T-cell subpopulation? To address this question we examined the LKB1 requirement for the proliferative responses of mature T cells. In these experiments we bypassed the LKB1 requirement for T-cell development by breeding mice expressing LKB1 floxed alleles to *CreER^T2^* mice that express a tamoxifen inducible Cre recombinase. In initial experiments, *CreER^T2^*LKB1^+/+^ and *CreER^T2^*LKB1^fl/fl^ peripheral T cells were polyclonally activated with CD3 Ab and maintained in exponential proliferation *in vitro* by the addition of IL-2. T cells were then treated with 4-hydroxytamoxifen (4OHT) to induce deletion of LKB1 in the *CreER^T2^*LKB1^fl/fl^ cells. The data (Fig. 5A) confirmed deletion of LKB1 following 4OHT treatment of *CreER^T2^*LKB1^fl/fl^ peripheral T lymphoblasts and revealed that LKB1 deletion stopped the proliferation of T cells and resulted in cell death (Fig. 5B and C). In further experiments, peripheral primary T cell isolated from *CreER^T2^*LKB1^+/+^ and *CreER^T2^*LKB1^fl/fl^ mice were cultured in IL-7 in the presence of 4OHT. Thereafter cells were stimulated with CD3/CD28 Ab coated beads to induce proliferation. The data in Fig. 5D show that 4OHT treated control lymphocytes from *CreER^T2^* mice (*CreER^T2^*LKB1^+/+^) gave a robust proliferative response following CD3/CD28 stimulation whereas *CreER^T2^*LKB1^fl/fl^ T cells did not. Both cell populations could be maintained with comparable viability when cultured in IL-7 (Fig. 5E) but the viability of the CD3/CD38 triggered LKB1-null cells were markedly reduced compared with the control LKB1 WT cells (Fig. 5E). These data show that LKB1 is required to support the survival of mature T cells when they are induced to proliferate.

### Discussion

The present results establish that LKB1 has critical functions during T-cell development. In the thymus LKB1 is required for the survival and differentiation of TCR-β selected T-cell progenitors. T-cell progenitors that successfully rearrange their TCR-β and express the pre-TCR complex normally proliferate rapidly prior to differentiation to the DP stage of thymus development. The present data now show that LKB1-null pre-T cells that have rearranged their TCR-β locus are unable to proliferate and undergo cell death rather than complete their normal program of development. The data also show that LKB1-null peripheral T cells are unable to proliferate and undergo cell death in response to CD3/CD28 stimulation. Activated T cells maintained in culture with IL-2 similarly fail to proliferate and die following LKB1 loss. It is well established that cells that fail to match energy production to energy demands die by apoptosis. LKB1 thus appears to coordinate the ability of T cells to match energy production to the energy requirements for the proliferative burst that accompanies TCR-β selection or the immune activation/cytokine-induced proliferation of peripheral T cells.

LKB1 phosphorylates and activates multiple members of the AMPK family including the α1 and α2 isoforms of AMPK, NUAK1-2, BRSK1-2, QIK, QSK, SIK, MELK and MARK1–4 kinases 21. There have been some studies to probe the role of individual members of this kinase family in T cells. For example, AMPK α1-null T cells have increased sensitivity to energy stress 32 and there are defects in immune homeostasis in mice that lack expression of another AMPK family kinase, MARK2/Par-1b 33. However, neither the AMPK α1 nor MARK2 mice showed any evidence for defective thymus development 32,33. This could mean that there is redundancy between members of the AMPK family in T cells. In this respect, redundancy between different serine/threonine kinases has been seen previously in T lymphocytes. For example, T cells express three isoforms of PKB/Akt and unveiling of the function of these kinases *in vivo* in the thymus required simultaneous deletion of all three isoforms 48. There is also redundancy between different isoforms of PI3K in T-cell progenitors such that deletion of both the p110 delta and p110 gamma catalytic subunits of PI3K is required to reveal the importance of PI3K in T-cell progenitors 49. The impact of LKB1 deletion *versus* deletion of individual AMPK family kinases is an indication that there is similar redundancy between different members of the AMPK family in T cells.

Previous studies have focused extensively on the role of PI3K and the serine/threonine kinases PKB/Akt in the control of T-cell metabolism 50.The present data showing that LKB1, a kinase that evolved to couple energy metabolism and cell differentiation, also has essential functions in T lymphocytes. It reveals that T cells use multiple pathways to deal with the metabolic stress of proliferation. Here it is important to note that these two kinase pathways function in quite different modes. In T cells, PKB/Akt activity is controlled directly by Ag receptors/costimulatory molecules and or cytokines and the level of Akt activity is determined by cellular levels of Phosphatidyinositol (3,4,5) tris phosphate and the activity of PDK1 and mTOR (mammalian target of rapamycin) 51. In contrast, LKB1 signaling pathways are not coupled directly to extracellular stimuli but rather act homeostatically in response to energy stress to restore energy balance. For example, LKB1 acts as a rheostat for cellular AMP/ATP ratios and phsophorylates and activates AMPK when cellular levels of ATP drop. This concept of how LKB1 works explains why it is not possible to explain the phenotype of LKB1-null T cells by the loss of a linear biochemical signaling pathway that is coupled to a single receptor. Rather LKB1 is integral to the control of T-cell proliferation irrespective of the proliferative stimulus.

## Materials and Methods

### Mice

Mice (5–7 weeks old) were maintained in SPF conditions at the University of Dundee under Home Office project license PPL60/3116. C57BL/6-Gt(ROSA)26Sor tm9(cre/Esr1)Arte mice carrying a transgene in the ROSA locus encoding for a ubiquitously expressed Cre recombinase/modified oestrogen receptor fusion protein *CreER^T2^*, were purchased from TaconicArtemis. LKB1^fl/fl^ mice were generated and bred as previously described 34. LKB1^fl/fl^ mice were crossed either to transgenic mice expressing Cre recombinase under the T-cell-specific Lck promoter 35 or to *CreER^T2^* mice. Genotyping was performed by PCR using genomic DNA isolated from ears. The presence of a WT or floxed LKB1 allele was detected using two primers: 5′-CCAGCCTTCTGACTCTCAGG-3′ and 5′-GTAGGTATTCCAGGCCGTCA-3′. PCR amplification of the WT or the floxed LKB1 alleles resulted in 200-bp and 250-bp PCR products, respectively. For the detection of Cre (either LckCre or *CreER^T2^*), the following primers were employed: 5′-AAATGGTTTCCCGCAGAACC-3′ and 5′-TAGCTGGCTGGTGGCAGATG-3′. Cre-mediated deletion of the floxed LKB1 allele was proved by PCR using primers: 5′-CAGGTTCAGCAGAATCAC-3′ and 5′-GGGAAGAAGGCGATATAAGG-3′.

### Flow cytometric analysis

Characterization of different thymocyte subpopulations was performed as described previously 7. Briefly, Ab conjugated to fluorescein isothiocyanate, phycoerythrin, allophycocyanin and biotin were obtained from either Pharmingen (San Diego, CA, USA) or eBioscience (San Diego, CA, USA). TriColour-conjugated Ab were obtained from Caltag (Burlingame, CA, USA). Cells were stained for surface expression of the following markers using the Ab in parentheses: CD4 (RM4-5), CD8 (53-6.7), CD25 (7D4), CD44 (IM7), CD98 (RL388), CD71 (C2), Thy1.2 (53-2.1), TCR-β (H57-597), B220 (RA3-6B2) and TCR/ (GL3). Cells were stained with saturating concentrations of Ab in accordance with the manufacturer's instructions. Data were acquired on either a FACS Calibur (Becton Dickinson, Franklin Lakes, NJ, USA) or a LSR1 flow cytometer (Becton Dickinson) using CellQuest software and were analyzed using either CellQuest (Becton Dickinson) or FlowJo (Treestar, San Carlos, CA, USA) software. Viable cells were gated according to their forward scatter and side scatter profiles. CD4^−^ and CD8^−^ DN subsets were gated by lineage exclusion (lineage) of all CD4^+^, CD8^+^ DP and SP cells and TCR-γ^+^. DN3 and DN4 were further defined as CD25^+^CD44^−^ and CD25^−^CD44^−^ thymocytes, respectively. Mature SP thymocytes were defined as Thy-1^+^, TCR-β^hi^ and positive for either CD4 or CD8 expression. Cell death was analyzed by staining with 7-aminoactinomycin D (5 μg/mL).

### Cell Purification

DN3 and DN4 thymocytes were purified by first depleting thymic populations of CD4^+^ and CD8^+^ cells using an AutoMACs magnetic cell sorter (Miltenyi Biotech, Auburn, CA, USA) before sorting to a purity greater than 95%, using a FACS VantageSE cell sorter (Becton Dickinson). Peripheral T lymphocyes (from spleen) were purified by AutoMACS magnetic cell sorter (Miltenyi Biotech) using mouse pan T-cell isolation kit.

### Intracellular TCR-β staining

Intracellular TCR-β staining was performed as described previously 10. Briefly: thymocytes were stained for cell surface markers to define DN3 and DN4 subsets. After fixation in 1% paraformaldehyde for 10 min at 25°C, cells were washed in PBS and permeabilized for 10 min at room temperature in saponin buffer (0.5% (weight/volume) saponin, 5% FBS and 10 mM HEPES, pH 7.4, in PBS) Permeabilized cells were incubated for 45 min with phycoerythrin-conjugated Ab to TCR in saponin buffer, were washed in saponin buffer and were analyzed on a FACS Calibur. Cell surface binding sites were blocked by biotinylated TCR and the specificity of staining was controlled by parallel staining with phycoerythrin-conjugated isotype-matched control Ab (Armenian hamster IgG2).

### Intracellular phospho-S6 staining

Intracellular phosphoS6 staining was performed as described previously 10. Thymocytes were treated with 20 nM rapamycin or were left untreated for 20 min at 37°C. Treatment of cells with rapamycin inhibits the activity of mTor and rapidly reverses S6 (Ser 235/236) phosphorylation. Phospho-S6 staining of rapamycin-treated cells thus provides an internal negative control as a standard for each sample. Cells were washed and stained with surface markers to define the DN3 and DN4 subsets, then were fixed in 0.5% paraformaldehyde for 15 min at 37°C, followed by 15 min in 90% methanol on ice. After fixation, cells were washed twice in BSA buffer (0.5% BSA in PBS), then blocked for 10 min at 25°C in BSA buffer. Cells were incubated for 30 min at 25°C with Ab to phospho-S6 (2211; Cell Signaling Technologies) in BSA buffer, then were washed and incubated for 30 min at 25°C with FITC-conjugated donkey IgG Ab to rabbit (Jackson ImmunoResearch). Samples were washed in BSA buffer and were analyzed on a FACS Calibur.

### Cell cycle analysis

The cellular DNA content of DN3 and DN4 thymocytes was analyzed on live cells, with Hoechst-33342 (Molecular Probes) staining. Cells were incubated for 1 h at 37°C in 5 μg/mL Hoechst-33342 in 2% FBS DMEM and then were surface stained for CD25, Thy-1 and lineage markers (including CD44). Samples were analyzed on an LSR1 flow cytometer eliminating doublets from the analysis.

### Quantitative RT-PCR

DN3 and DN4 Cells (0.5×10^6^ per sample) were lysed for RNA isolation using the QIAshredder homogenizer (QIAGEN). RNA was purified with the RNeasy Mini, RNA isolation kit (QIAGEN) following the manufacturer's protocol and including an on-column DNase digestion step with the RNase-free DNase set (QIAGEN). cDNA was generated using the iScript™ cDNA synthesis kit (BIO-RAD). Real-time PCR reactions were performed in a 20 μL mixture containing 1× iQ™ SYBR Green supermix (BIO-RAD), 1 μL of cDNA preparation and 0.4 μM forward and reverse primers. Real-time quantitations were performed using the BIO-RAD iCycler iQ system and using 18S rRNA as an internal control. The primer sequences for the PCR were as follows

**Table:** 

Hes1	Forward 5′-ACCTTCCAGTGGCTCCTC-3′
	Reverse 5′-TTTAGTGTCCGTCAGAAGAGAG-3′
CD2	Forward 5′-GTATGAGGTCTTAGCAAACGGATC-3′
	Reverse 5′-TGTGCCATACACCATTACATTATAGG-3′
Nur77	Forward 5′-CCTGTTGCTAGAGTCTGCCTTC-3′
	Reverse 5′-CAATCCAATCACCAAAGCCACG-3′
Deltex1	Forward: 5′-CTTCTGCTACCTCATCTACTTCAA-3′
	Reverse: 5′-GGAATGGAGCCGACAGTGAG-3′
18S	Forward 5′-ATCAGATACCGTCGTAGTTCCG-3′
	Reverse 5′ TCCGTCAATTCCTTTAAGTTTCAGC-3′

### Cell culture

OP9 bone marrow stromal cells expressing OP9-DL1 and control OP9 cells were a gift from Juan Carlos Zúñiga-Pflücker (Toronto, Canada) 46. OP9 cells were maintained in αMEM supplemented with 50 μM 2-mercaptoethanol, 100 U/mL penicillin, 1 mg/mL streptomycin and 20% heat-inactivated FBS. DN thymocytes were co-cultured on OP9-DL1 monolayers for times indicated in figure legends in the presence or absence of 5 ng/mL of IL-7 as indicated. For harvesting, thymocytes were filtered through 50 μm filters to remove OP9-DL1 cells before developmental progression of T lineage cells was assessed. For activation of primary T cells spleens or lymph nodes were removed from *CreER^T2^*LKB1^+/+^ and *CreER^T2^*LKB1^fl/fl^ mice, disaggregated and red blood cells were lysed. Cells were cultured in RPMI-1640 medium containing l-glutamine (Invitrogen), 10% v/v heat-inactivated FBS (Gibco), 50 μM β-mercaptoethanol (Sigma) and penicillin–streptomycin (Gibco).

Single-cell suspensions from lymph node preparations cultured at 5×10^6^ cells *per* mL. Cells were treated with 0.6 μM 4OHT in the presence of 5 ng/mL IL-7 for 4 days. Thereafter cells were either stimulated with CD3/CD28 Ab-coated beads (Dynabeads Mouse CD3/CD28T cell expander, Invitrogen) or left in 5 ng/mL IL-7 for 3 days.

For the generation of lymphoblasts, mouse CD8^+^ T cells were cultured for 48 h in the presence of a CD3 Ab (5 μg/mL; 145-2C11; R&D Systems) to trigger the TCR. Thereafter the cells were washed and maintained in exponential proliferation with 20 ng/mL IL-2 (Chiron) for another 2 days. Then the cells were treated with 0.4 μM 4OHT for 3 days. The 4OHT was washed and the cell proliferation was investigated in the presence of IL-2.

### Western blot analysis

Sorted DN4 thymocytes or peripheral T lymphoblasts were lysed on ice in NP-40 lysis buffer (50 mM HEPES (pH 7.4), 75 mM NaCl, 1% Nonidet P-40, 10 mM sodium fluoride, 10 mM iodoacetamide, 1 mM EDTA, 40 mM β-glycerophosphate, protease inhibitors, 100 μM sodium orthovanadate). Lysates were centrifuged at 1600×*g* for 15 min at 4°C. Protein samples were separated by SDS NuPAGE 4–12% Bis-Tris PAGE (Invitrogen) transferred to nitrocellulose membrane and detected by Western blot analysis using standard techniques. The phosphorylated AMPK (phospho-172Thr-AMPK Rabbit mAb (Cell Signaling Technology) and α1 AMPK antisera were used as described 31. The LKB1 Ab from Cell Signaling Technology was used to probe for LKB1 expression.

### Statistical analysis

Statistical analyses were performed using GraphPad Prism 4.00 for Macintosh, GraphPad Software. A non-parametric Mann–Whitney test was used where the number of experiments performed was not sufficient to prove normal distribution. When comparing gene expression between LckCre^+^LKB1^+/+^ and LckCre^+^LKB1^fl/fl^ animals, a Student's *t*-test was used with the theoretical mean of LckCre^+^LKB1^+/+^ sample set to 1. Differences were considered as significant if *p*<0.05.
